# Sicilian Semi- and Supercentenarians: Age-related NK Cell Immunophenotype and Longevity Trait Definition

**DOI:** 10.37825/2239-9747.1041

**Published:** 2023-10-17

**Authors:** Mattia E. Ligotti, Giulia Accardi, Anna Aiello, Anna Calabrò, Calogero Caruso, Anna M. Corsale, Francesco Dieli, Marta Di Simone, Serena Meraviglia, Giuseppina Candore

**Affiliations:** a Laboratory of Immunopathology and Immunosenescence, Department of Biomedicine, Neurosciences and Advanced Diagnostics, University of Palermo, Palermo, Italy; b Central Laboratory of Advanced Diagnosis and Biomedical Research, University Hospital “P. Giaccone”, Palermo, Italy; c Department of Biomedicine, Neurosciences and Advanced Diagnostics, University of Palermo, Palermo, Italy

**Keywords:** Immune ageing, Immunophenotype, Longevity, Natural killer cells, Semi-supercentenarians, Supercentenarians

## Abstract

The immune system of semi- and supercentenarians (i.e., the oldest centenarians) is believed to have peculiar characteristics that enable them to reach extreme longevity in a relatively healthy state. Therefore, in previous papers, we investigated, through flow cytometry, variations in the percentages of the main subsets of Tαβ and Tγδ cells in a Sicilian cohort of 28 women and 26 men (age range 19–110 years), including 11 long-living individuals (>90 years old) and 8 oldest centenarians. These investigations suggested that some observed immunophenotypic changes may contribute to the extreme longevity of the oldest centenarians. In the present study, to further characterize the immunophenotype of the oldest centenarians, we examined the percentages of Natural Killer (NK) cells identified as CD3−CD56 + CD16+ in the previously described Sicilian cohort. We found a highly significant increase in NK cell percentages with age. When stratified by gender, this significant increase with age was maintained in both sexes, with higher significance observed in males. Our findings on NK cells, together with the previously obtained results, discussed in the context of the literature, suggest that these changes are not unfavourable for centenarians, including the oldest ones, supporting the hypothesis that immune aging should be considered as a differential adaptation rather than a general immune alteration. These adapted immune mechanisms allow the oldest centenarians to successfully adapt to a history of insults and achieve remarkable longevity.

## 1. Introduction

The immune system of semi- and supercentenarians (*i.e*., oldest centenarians) is believed to have peculiar characteristics that enable them to reach extreme old age in a relatively healthy state [1–3]. Therefore, the immunophenotype of the oldest centenarians could provide important information about their ability to adapt to factors associated with immune changes, including aging itself and chronic cytomegalovirus (CMV) infection known to affect lymphocyte subset values [4,5]. In a previous study [4], we investigated the percentage and absolute numbers of immune cell subsets, focusing on Tαβ cells, and pro-inflammatory parameters in a Sicilian cohort of 28 women and 26 men (age range 19–110 years), including 11 long-living individuals (LLIs >90 < 105 years) and 8 oldest centenarians (105–110 years), using flow cytometry. We observed variability in hallmarks of immunosenescence related to age and CMV serological status [6]. In a subsequent study, we investigated the percentage variations of the two main subsets of Tγδ cells, Vδ1 and Vδ2, and their functional subsets using markers defining Tαβ cells, *i.e*., CD27 and CD45RA, in all subjects previously analysed for Tαβ immunophenotypes in the same blood samples, still using flow cytometry [7]. The results of these two studies have suggested that some changes observed in the immunophenotypes of the oldest centenarians might represent an immune mechanism by which the oldest centenarians successfully adapt to a history of insults and achieve longevity.

To gain insight into the immunophenotypes of the oldest centenarians, in the present paper, we have analysed, in the previously described Sicilian cohort, the percentages of Natural Killer (NK) cells identified as CD3−CD56 + CD16+, markers that recognize 90% of circulating NK cells [8], known to be mainly responsible for natural cytotoxicity by releasing cytoplasmic granules containing perforin and granzymes B [9].

## 2. Materials and methods

For the present paper, we have studied 54 Sicilians aged between 19 and 110 years, enrolled between 2020 and 2022, selected because of the absence of health issues (LLIs and the oldest centenarians were relatively healthy). Detailed study design and participant recruitment have been previously described [5,10]. The cohort included adult people (n = 20; 10 males and 10 females; years/months range 19.5–63.6); Older people (n = 15; 8 M and 7 F; range 68.5–87.3); LLIs (n = 11; 7 M and 4 F; range 93.3–104.7); Oldest centenarians (n = 8; 1M and 7 F; range 105.7–110.3). All LLIs and oldest centenarians were CMV-seropositive, whereas were CMV-seropositive 78% of older and 63% adult people [5].

The Ethics Committee of Palermo University Hospital had approved the study protocols (Nutrition and Longevity, No. 032017). All the studies had been conducted in accordance with the Declaration of Helsinki and its amendments. All study participants (or their children) had given their written informed consent prior to enrolment.

**Fig. 1:**
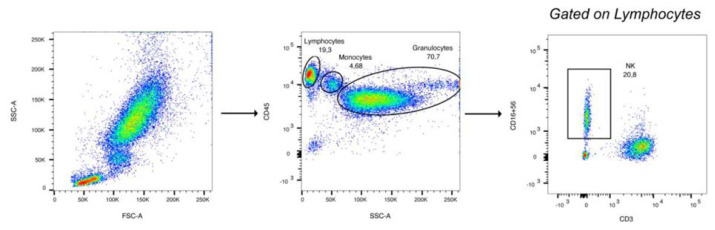
Gating strategy for the identification of NK lymphocytes (CD16 + CD56 + CD3−). For explanation see text.

Flow cytometry analysis was conducted on fresh whole blood, after red blood cell lysing, using the following antibodies from BD Bioscience: CD3−FITC, CD16/56-PE, and CD45−PerCP/Cy5.5. To perform this analysis was used FACS Canto (BD) at the Central Laboratory of Advanced Diagnosis and Biomedical Research, “P. Giaccone” University Hospital, Palermo. To identify lymphocytes, monocytes, and granulocytes, forward-scatter (FSC) and side-scatter (SSC) were checked, and further characterization was done on the SSC/CD45 dot-plot. The gating strategy applied is illustrated in Fig. 1. Firstly, the lymphocytes region was set in the CD45 positive and SSC-A low gate, and then events were gated in the CD3 negative and CD16/56 positive region. The results were expressed as a fraction of the parental gated population (lymphocytes) and presented as percentages in the graphs.

To analyse the percentages of NK cells, statistical analysis was performed with GraphPad Prism version 9.3.1 (GraphPad Software, San Diego, CA, USA). Correlation between the percentage of NK cells and age in all individuals and males and females was examined using simple linear regression analysis. Figures were plotted as scatter plots with a linear regression line and 95% confidence bands. Analysis of NK cells between age groups was performed by one-way ANOVA test. For each statistical analysis, only *p*-values <0.05 were considered significant.

## 3. Results and Discussion

**Fig. 2:**
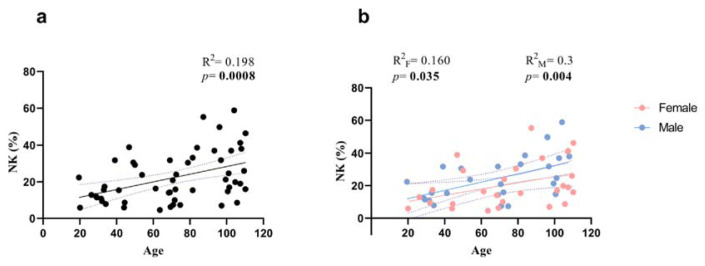
Age-related changes in NK cells (CD16 + CD56 + CD3−). Linear regression analysis showing the relationship between NK cells and age in all individuals (n = 54) (black line) (a), males (n = 26) (blue line) and females (n = 28) (b) (pink line). Each point represents data from an individual healthy donor. The coefficient of determination and p-values are shown on the graphs. F = female; M = male; NK= Natural Killer cells; R^2^=R squar

**Fig. 3:**
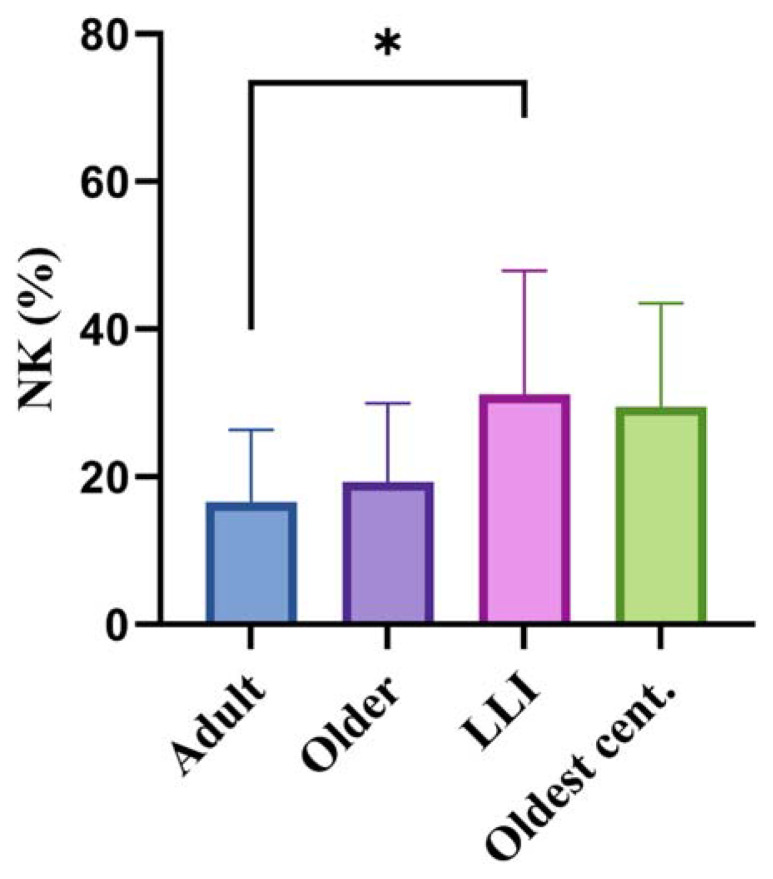
NK cells (CD16 + CD56 + CD3−) percentages divided into four age groups for comparative analysis. Column bar graphs showing differences between the mean of the values of NK cells from each group obtained by one-way ANOVA test. * p-value = 0.0119.

We analysed the frequency of NK cells, referred to as CD3−CD16 + CD56+, in the peripheral blood of all subjects involved in the study and we found a very significant increase age-related in NK percentage ( *p* = 0.00008; Fig. 2a). Following gender stratification, the significant increase with age was maintained in both sexes with a higher significance in males (Females *p* = 0.035; Males *p* = 0.004; Fig. 2b). By analysing the data by one-way Anova test (Fig. 3) the age-related increment of NK cells was significant in adults vs. LLIs (p < 0.0119), while in adults vs. oldest centenarians the significance was bordeline (p = 0.0745).

The absolute number of CD56 + CD16+ NK cells has been reported variably as maintained, increased, or decreased in older individuals and consistently increased in centenarians [4,6,11]. Our results not only confirm the increase of CD56 + CD16+ NK cells in centenarians but also extend these findings to NK cells of semi- and supercentenarians, which, to the best of our knowledge, have never been studied before. As with other lymphocyte subsets, the data from the oldest centenarians show great heterogeneity, with some individual displaying a remarkable increase in NK cell values, while other individuals show values similar to those of younger subjects [5,7]. That is likely due to individual unique immunological history, i.e., immunobiography [12]. Finally, the degree of NK cell increase, as measured by significance, was slightly lower in females compared to males.

Ligotti et al. [5], observed that the oldest centenarians exhibited the lowest percentages of naive T cells, due to their age, and the highest percentages of effector memory T cells re-expressing CD45RA (T_EMRA_), based on their CMV status, along with elevated levels of pro-inflammatory parameters in the serum, although their averages were lower than those of the remaining donors over the age of 90. However, some of them showed percentages of naive T cells and T_EMRA_ CD8 cells, which are thought to be markers of exhaustion/pro-inflammatory responses, comparable to those of younger individuals. However, data obtained on inflammatory markers, T_EMRA_, and CMV seropositivity in centenarians, discussed in light of the most recent research, suggest that these changes may not be unfavourable for centenarians, particularly the oldest ones. In fact, CMV is responsible for a large subset of T effector memory virus-specific cells, and many of these are T_EMRA_. These cells are perfectly equipped to control the virus without further T-cell expansion [13,14].

In a subsequent paper on Tγδ [7], the most interesting data concerned the T_EMRA_ cells, which were significantly increased in Vδ1 cells, with the highest values observed in the oldest centenarians, albeit with considerable heterogeneity. Our findings on γδ T_EMRA_ cells, discussed in the context of the literature, suggest that these changes are not unfavourable for centenarians, including the oldest ones. Indeed, *in vivo* Tγδ T_EMRA_ expansion is associated with a reduced risk of cancer onset or leukaemia recurrence as well as with clearance of CMV infection, in allogeneic stem cell recipients as well as in kidney transplant patients respectively [15].

In the present paper, we have shown a highly significant age-related increase of CD56 + CD16+ NK cells with the highest values observed in the oldest centenarians, although with considerable heterogeneity. It is intriguing that it has been demonstrated that the percentage of circulating CD16 + CD56+ NK cells was negatively correlated with the occurrence of colorectal cancer and its staging [16]. Furthermore, NK cells, together with macrophages, have been shown to play a major role in senescent cell, known to drive organismal ageing [17], immune surveillance. Their activating receptors are able to promptly recognize stressed cells, hence that makes NK cells peculiar in their role of sentinels of senescent cells [18]. Both these aspects might be valuable mechanism to reduce cancer incidence (or severity) and senescence burden respectively, hence favouring the achievement of longevity.

However, our study has some limitations: i) the cross-sectional nature of the data, which is common in most immunophenotypic studies in the literature; ii) the inability to determine the role of gender because differences may have been influenced by gender disparity in favour of oldest female centenarians (in the cohort there is only a male oldest centenarian); iii) it is impossible to separate the effects of age from those of CMV as Sicilian people over 90 years old is CMV positive; however, it has been reported that ageing, but not CMV seropositivity, affects NK cell subsets defined by CD56 and CD16 [19]; iv) another possible limitation could be the lack of functional data of different NK subsets, as well as the lack of analysis of NK CD16-cells that constitute 10% of NK subset in adult people [8], described as secreting chemokines and cytokines [20], and decrease with age [4]. However, the aim of the present study was to investigate the variations of CD16 + CD56+ NK cells, known to increase in centenarians, in a cohort comprising oldest centenarians to complement our previous studies on Tαβ and Tγδ cells variations.

In conclusion, our results regarding Tαβ, Tγδ cells and those presented in the present study on NK reinforce the hypothesis that immune ageing should not be considered as a general immune alteration but rather as a differential adaptation. Thus, the increase in T_EMRA_ CD8+ and Vδ1, as well as CD16 + CD56 NK cells, would represent immune mechanisms by which centenarians, in particular the oldest ones, successfully adapt to a history of antigenic burden, thus achieving longevity.
